# Temporal Trends in Chorioamnionitis by Maternal Race/Ethnicity and Gestational Age (1995–2010)

**DOI:** 10.1155/2013/906467

**Published:** 2013-04-04

**Authors:** Michael J. Fassett, Deborah A. Wing, Darios Getahun

**Affiliations:** ^1^Division of Maternal-Fetal Medicine, Department of Obstetrics-Gynecology, West Los Angeles Kaiser Permanente Southern California Medical Group, Los Angeles, CA 90034, USA; ^2^Division of Maternal-Fetal Medicine, Department of Obstetrics-Gynecology, University of California, Irvine, CA 92868, USA; ^3^Department of Research & Evaluation, Kaiser Permanente Southern California, Pasadena, CA 91101, USA; ^4^Department of Obstetrics and Gynecology, University of Medicine and Dentistry New Jersey, New Brunswick, NJ 08901, USA

## Abstract

*Objective*. To characterize trends in chorioamnionitis (CAM) by maternal race/ethnicity and gestational age. *Study Design*. We examined trends in CAM from 1995–2010 among singleton births in all Kaiser Permanente Southern California hospitals (*n* = 471,821). Data were extracted from Perinatal Service System and clinical utilization records. Gestational age- and race/ethnicity-specific biannual diagnosis rates were estimated using the Poisson regression after adjusting for potential confounding factors. *Results*. Overall diagnosis rates of CAM increased from 2.7% in 1995-1996 to 6.0% in 2009-2010 with a relative increase of 126% (95% confidence intervals [CI] 113%–149%). From 1995-1996 to 2009-2010, CAM increased among the Whites (1.8% to 4.3%, *P*-value for trend <.001), Blacks (2.2% to 3.7%, *P*-value for trend <.001), Hispanics (2.4% to 5.8%, *P*-value for trend <.001), and Asian/Pacific Islanders (3.6% to 9.0%, *P*-value for trend <.001). The adjusted relative percentage change in CAM from 1995-1996 to 2009-2010 was for Whites [preterm 21% (9%–78%), term 138% (108%–173%)], for Blacks [preterm 24% (−9%–81%), term 62% (30%–101%)], for Hispanics [preterm 31% (3%–66%), term 135% (114%–159%)], and for Asian/Pacific Islanders [preterm 44% (9%–127%), term 145% (109%–188%)]. *Conclusion*. The findings suggest that CAM diagnosis rate has increased for all race/ethnic groups. This increase is primarily due to increased diagnosis at term gestation.

## 1. Introduction

Chorioamnionitis, an infection and inflammation of the maternal and fetal interface, is arguably the most important cause of preterm birth and infant morbidity. Despite advancements in diagnosis and treatment, chorioamnionitis and its complications remain major public health concern in the United States. It has been estimated that about 10 percent of all pregnancies are complicated by chorioamnionitis [1, 2]. Documented immediate and long-term sequelae of chorioamnionitis include fetal mortality [3], preterm premature rupture of membranes [4], neonatal intensive care admission [5], bronchopulmonary dysplasia [6, 7], and cerebral palsy [8]. Most importantly, chorioamnionitis is responsible for approximately half of all preterm births [9–11]. Prevalence varies with race/ethnicity and is higher in non-Whites than Whites [12].

The most common route of infection is ascending microbial invasion of the amniotic cavity from upper genital tract [13, 14]. Inflammatory processes at sites remote from the female genital tracts are also described as important sources of infection [15, 16].

There is a gap in knowledge about the recent trends in chorioamnionitis diagnosis rate and the modifying role of maternal race/ethnicity. This gap coupled with its association with preterm birth and the known differential risk of preterm labor based on race/ethnicity led us to speculate that there may be differential temporal trends in chorioamnionitis diagnosis rates based on maternal race/ethnicity and gestational age at delivery. 

## 2. Materials and Methods

The cohort for this study is comprised of all women with a singleton birth at ≥20 weeks gestation who delivered in a KPSC hospital from 1995 to 2010. For these women, we matched the Perinatal Services System (PSS) record, which contained information from the infant's birth certificate (maternal demographics, behavioral characteristics, complications of labor and delivery, and fetal/infant birth outcomes from other sources) with the hospital inpatient and outpatient physicians encounters records, which included more detailed information on maternal medical and obstetrical history and fetal and infant outcomes. 

International Classification of Diseases, Ninth Revision Clinical Modification (ICD-9-CM) codes “762.7” and “658.4x” were used to identify the clinically diagnosed chorioamnionitis. We validated the accuracy of the ICD-9-CM coding by comparing it with diagnoses abstracted from a random sample of 400 medical records. Pregnancies resulting in preterm births or low birthweight were oversampled to ensure adequate number of subjects with chorioamnionitis.After adjusting for sampling fractions, the estimated sensitivity, specificity and positive and negative predictive values for chorioamnionitis were 100%, 99%, 92%, and 100%. These findings support the validity of the diagnosis codes in our study. Gestational age, expressed in completed weeks, was based on the clinical estimates of gestational ages contained in electronic medical records. Maternal race/ethnicity was based on information from the infant birth certificate and categorized as non-Hispanic White (White), non-Hispanic Black (Black), Hispanic, and Asian/Pacific Islander. 

Of 505,911 pregnancies from 1995 to 2010 we excluded multiple pregnancies (*n* = 15,798), early pregnancy terminations (*n* = 5,306), pregnancies delivered at <20 weeks of gestation (*n* = 1,641), and women with “other” or missing race/ethnicity. We decided to exclude women of “other” race/ethnicity from all analyses due to the small number of such women (11,345; 2%). After these exclusions, 471,821 women remained for analysis.

We estimated the overall and race/ethnicity-specific annual diagnosis rates of chorioamnionitis per 100 singleton births using the Poisson regression. For this, the yearly count of chorioamnionitis diagnosis was the outcome variable and year of diagnosis was the independent variable, adjusting for potential confounding factors listed in Table 1. We also examined time trends by comparing event rates in the earliest (1995-96) versus most recent (2009-10) periods and quantified their 95% confidence interval (CI). In order to assess race/ethnicity disparity in the diagnosis of chorioamnionitis, we compared rates for each of the three racial/ethnic groups to those of White women. Variables considered as potential confounders included maternal age (<20, 20–29, 30–34, and ≥35 years), education (<12, 12, and ≥13 years of completed schooling), median family household income based on census tract of residence (<$29,999, $30,000–$49,000, $50,000–$69,999, $70,000–$89,999, and ≥$90,000), prenatal care (care initiated in the first trimester versus late or no prenatal care), smoking during pregnancy (yes/no), and parity. Finally, because the recent trends in chorioamnionitis diagnosis may be modified by the rising trends in induction of labor, we performed subanalyses after stratifying the data by no induction, medically indicated induction, and elective induction of labor categories. Elective induction of labor was defined as initiation of labor performed in the absence of any of the medical or obstetrical indication recommended by ACOG [17].

All analyses were performed using SAS 9.2 (SAS institute, Cary, NCUSA).The study was approved by the Kaiser Permanente Southern California (KPSC) Institutional Review Board.

## 3. Results 

The overall chorioamnionitis rate in women delivering in all KPSC hospitals has risen from 3.2 percent in 1995-1996 to reach its highest level yet in 2009-2010 of 5.6 percent, with a relative increase of 79% (95% confidence interval (CI) 69%, 90%). Women with chorioamnionitis tend to be younger than those without chorioamnionitis. Race/ethnicity varied between women with chorioamnionitis and those without chorioamnionitis; in particular, there were more Hispanics or Asian/Pacific Islanders among women with chorioamnionitis (Table 1). Women with chorioamnionitis tended to be nulliparous, had more formal education, and were more likely to have their labor induced than women without chorioamnionitis.

Table 2 shows the recent trends in chorioamnionitis diagnosis between 1995-1996 and 2009-2010 based on race/ethnicity adjusted for maternal age, education, median household income, parity, prenatal care, and smoking during pregnancy. Chorioamnionitis diagnosis rates have increased by 141% (95% CI 109%, 178%; *P* value for trend <.001) among White women, 145% (95% CI 122%, 171%; *P* value for trend <.001) among Hispanic women, and 151% (95% CI 113%, 197%; *P* value for trend <.001) among Asian/Pacific Islander women. The increase in chorioamnionitis diagnosis was lowest among Black women: 66% (95% CI 33%, 108%; *P* value for trend  .001). 

The relative (percent) changes in chorioamnionitis rates between the earliest and the most recent years based on maternal race/ethnicity and gestational age categories are shown in Table 3. After adjusting for maternal age, education, prenatal care, smoking, and median household income, we observed significant changes in chorioamnionitis rates from 1995-96 to 2009-10 among the Whites (preterm birth 21%, 95% CI 9%, 78% and term birth 138%, 95% CI 108%, 173%), among Hispanics (preterm birth 31%, 95% CI 3%–66% and term birth 135%, 95% CI 114%, 159%), and among Asian/Pacific Islanders (preterm birth 44%, 95% CI 9%, 127% and term birth 145%, 95% CI 109%, 188%). We observed a nonsignificant percent change in the diagnosis of chorioamnionitis for the Blacks at a preterm birth (24%, 95% CI −9%, 81%) and significant percent change at term birth (62%, 95% CI 30%, 101%).

**Figure 1:**
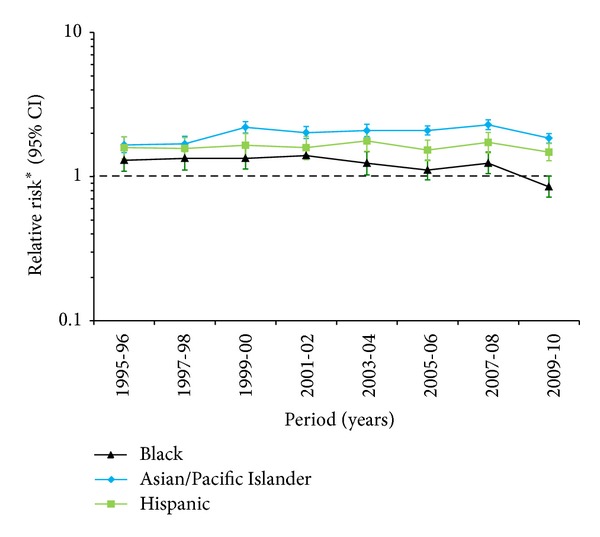
Race/ethnic disparity in rates of chorioamnionitis (CAM). ∗Adjustments were made for maternal age, education, median household income, parity, prenatal care, smoking during pregnancy, and induction of labor.

Figure 1 describes the race/ethnicity disparity in the diagnosis of chorioamnionitis among studied pregnant population, using White race as the reference. Hispanic and Asian/Pacific Islander women have significantly higher chorioamnionitis rates than White women throughout the entire study period. However, from 2003 to 2010, the gap in the diagnosis of chorioamnionitis has narrowed for the Blacks.

**Figure 2:**
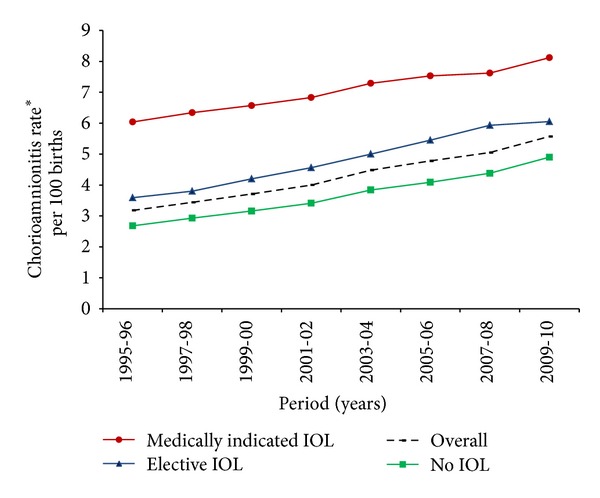
Temporal trends in the rate of chorioamnionitis (CAM) based on induction of labor subtypes. IOL: induction of labor. ∗Adjustments were made for maternal age, race/ethnicity, education, median household income, parity, prenatal care, and smoking during pregnancy.

During the study period, the rate of induction of labor has increased by 46% between 1995-1996 (21.1%) and 2009-2010 (30.7%) for all singleton births. In order to assess the effect of induction of labor on the rate of chorioamnionitis, we repeated the analysis after stratifying the data by indication of labor subtypes (indicated, elective, and spontaneous). Women who had a medically induced labor consistently had the highest chorioamnionitis diagnosis followed by women who had elective induction of labor (Figure 2). 

## 4. Discussion

The rate of chorioamnionitis among women with singleton pregnancies delivered in the KPSC hospitals increased by 79% between 1995-1996 and 2009-2010. Our data further showed disparity in rate of chorioamnionitis by maternal race/ethnicity, which is not explained by maternal sociodemographic, behavioral, and perinatal factors. There is a staggering increase in the rate of chorioamnionitis at preterm gestation among all but Black race/ethnicity groups. While there are temporal trends in chorioamnionitis rates at term gestation among women from all racial/ethnic groups, there was significant heterogeneity in the magnitude. While Hispanic and Asian/Pacific Islander women have significantly higher rates of chorioamnionitis diagnosis, Black women have rates of diagnosis that are comparable to those of their White counterparts. 

The clinical explanation for our observation in the increase in chorioamnionitis at term is unclear.We hypothesized that the increasing use of labor induction, as a proxy for chorioamnionitis risk factors such as longer labors and frequent cervical exams [18, 19], was not found to be a significant confounding factor.Though Asian/Pacific Islander women have been shown to have significantly longer second stage and rates of prolonged second stage as compared to White women, the absolute prolongation of approximately five minutes does not seem to account for our observed increase in rates of chorioamnionitis [20]. Changing intrapartum antibiotic use after the Centers for Disease Control 2002 Perinatal GBS Prevention Guidelines may temporally be consistent with our observed increase in chorioamnionitis; however overall rates of early onset neonatal sepsis have remained unchanged [21].

While chorioamnionitis may be thought of as more of a risk for preterm infants, significant morbidity has been reported in term births complicated by chorioamnionitis. Alexander et al. [22] reported on more than 5000 term infants born to women with chorioamnionitis and reported significant association with intubations in the delivery room, neonatal pneumonia, and neonatal sepsis. Chorioamnionitis in term infants is a significant risk factor for development in cerebral palsy. In a nested cohort study of more than 230,000 singletons born at 36 weeks or more, chorioamnionitis was found to contribute to 11% of cerebral palsy cases, with an OR of 4.1 for developing cerebral palsy after a diagnosis of chorioamnionitis [8]. In addition to increasing neonatal morbidity, term chorioamnionitis increases maternal morbidity as well.The Maternal-Fetal Medicine Network compared 1965 women with chorioamnionitis to 14685 women who were not. Maternal risks of uterine atony, blood transfusion, pelvic abscess, thromboembolism, and wound complications were increased with chorioamnionitis [23].Given our results showing an overall increase in chorioamnionitis at term being responsible for the temporal increase in chorioamnionitis, more term infants are at risk for infection-related morbidity.

The strength of this study is that it represents a large, population-based, racially, ethnically, and socioeconomically diverse group of women from Southern California for whom we have comprehensive information from multiple clinical record sources. To our knowledge there is no data in the literature on the recent population trends of chorioamnionitis. In particular, findings of this study demonstrated heterogeneity in the prevalence of chorioamnionitis among studied racial groups. Nevertheless, some limitations should be acknowledged. The coding of behavioral risk factors such as smoking during pregnancy may not always be reliable and, additionally, these behaviors may be underreported; thus the potential for residual confounding remains. Because we do not have data on antibiotic use during pregnancy by the timing of pregnancy (antepartum or intrapartum) until the electronic medical record (EMR) has been fully implemented across the Kaiser Permanente healthcare system in 2008, we were unable to investigate the impact of the Centers for Disease Control 2002 Perinatal GBS Prevention Guidelines for intrapartum antibiotic use on the observed trends.

## 5. Conclusions

The findings of this study demonstrated significant variations in the temporal trends of chorioamnionitis by race/ethnicity. Chorioamnionitis diagnosis rates increased for women of all race/ethnic groups, both at preterm and term gestation. The preponderance of chorioamnionitis at term gestation among Hispanic and Asian/Pacific Islander women appears responsible for the observed disparity in chorioamnionitis. 

## Figures and Tables

**Table 1:** Distribution of maternal characteristics based on chorioamnionitis (CAM) status.

	No CAM	CAM
Characteristics	(*n* = 452,393)	(*n* = 19,428)
	%	%
Maternal age (years)∗		
<20	6.1	7.4
20–29	45.9	48.0
30–35	28.2	26.9
≥35	19.8	17.6
Race/ethnicity∗		
Non-Hispanic White	27.0	20.2
Non-Hispanic Black	10.4	8.7
Hispanics	51.6	53.1
Asian/Pacific Islanders	11.0	18.1
Maternal education (years)		
<12	13.9	10.4
12	29.5	25.4
≥13	53.3	60.0
Missing	3.3	4.2
Household income^∗‡^		
<$30,000	6.0	6.2
$30,000–$49,999	27.7	28.2
$50,000–$69,999	29.5	30.0
$70,000–$89,999	19.6	19.3
≥$90,000	17.2	16.3
Late or no prenatal care∗	14.8	13.0
Smoking during pregnancy	9.5	10.5
Parity∗		
0	38.5	72.0
1	33.2	18.1
2	18.1	6.5
≥3	10.3	3.4
Induction of labor∗	24.0	39.8

∗Differences between CAM and No-CAM by maternal characteristics were statistically significant (*P* < .001); ^‡^Median household income based on census tract information.

**Table 2:** Chorioamnionitis diagnosis rate per 100 singleton births by Race/ethnicity in KPSC Hospitals, 1995–2010.

Period	Total births	Overall rate (%)	Maternal Race/Ethnicity			
			White	Black	Hispanic	Asian/PI
1995-96	51495	2.67	1.79	2.20	2.38	3.59
1997-98	62432	2.98	2.01	2.27	2.63	4.08
1999-00	63971	3.32	2.28	2.51	2.93	4.66
2001-02	60007	3.69	2.59	2.78	3.37	5.45
2003-04	57511	4.30	3.15	3.07	4.07	6.39
2005-06	57062	4.78	3.51	3.36	4.56	7.13
2007-08	60682	5.23	3.84	3.58	5.04	7.93
2009-10	58661	5.96	4.32	3.65	5.82	9.02

*P* for trend from 1995 to 2010		<.001	<.001	.001	<.001	<.001

Relative increase % (95% CI), 2009-10 versus 1995-96		126 (113, 149)	141 (109, 178)	66 (33, 108)	145 (122, 171)	151 (113, 197)

Rates are expressed in percent; Adjustments were made for maternal age, education, median household income, parity, prenatal care, and smoking during pregnancy; Asian/PI: Asian and Pacific Islanders; CI: confidence interval.

**Table 3:** Chorioamnionitis rate per 100 singleton births by race/ethnicity at term gestation in KPSC Hospitals, 1995–2010.

	Chorioamnionitis rate at preterm (<37 wks) and term (37–42 wks) gestations							
Period	White		Black		Hispanic		Asian/PI	
	<37 wks	37–42 wks	<37 wks	37–42 wks	<37 wks	37–42 wks	<37 wks	37–42 wks
1995-96	5.02	1.87	7.29	2.29	5.93	2.54	5.87	3.84
1997-98	5.18	2.12	7.36	2.43	6.11	2.84	6.07	4.41
1999-00	5.38	2.39	7.68	2.60	6.36	3.12	6.68	5.02
2001-02	5.47	2.66	7.87	2.78	6.62	3.49	6.81	5.75
2003-04	5.74	3.11	8.35	2.98	7.13	4.09	7.21	6.58
2005-06	6.21	3.48	8.83	3.21	7.50	4.57	7.70	7.30
2007-08	5.99	3.89	8.88	3.55	7.53	5.08	7.74	8.22
2009-10	6.00	4.45	8.90	3.69	7.73	5.98	8.39	9.41

*P* for trend	<.001	<.001	.049	<.001	<.001	<.001	<.001	<.001

Relative increase % (95% CI), 2009-10 versus 1995-96	21 (9, 78)	138 (108, 173)	24 (9, 81)	62 (30, 101)	31 (3, 66)	135 (114, 159)	44 (9, 127)	145 (109, 188)

Rates are expressed in percent; Adjustments were made for maternal age, education, median household income, parity, prenatal care, and smoking during pregnancy; Asian/PI: Asian and Pacific Islanders; CI: confidence interval.
